# Development and Validation of Two Short Forms of the Managing the Emotions of Others (MEOS) Scale

**DOI:** 10.3389/fpsyg.2018.00974

**Published:** 2018-06-14

**Authors:** Elizabeth J. Austin, Donald H. Saklofske, Martin M. Smith

**Affiliations:** ^1^Department of Psychology, University of Edinburgh, Edinburgh, United Kingdom; ^2^Department of Psychology, University of Western Ontario, London, ON, Canada

**Keywords:** MEOS scale, interpersonal emotional management, emotional intelligence, “dark side” of EI, short form

## Abstract

The 58-item MEOS assesses managing the emotions of others, a component of trait emotional intelligence (EI). Managing another person's emotions can be used with the intention of helping the target but also in a strategically manipulative manner; the subscales of the MEOS cover both these aspects of emotion management. In order to allow researchers to access shorter versions of the MEOS for use in studies where administering the full-length scale is not feasible, two short forms of the MEOS with six (MEOS-SF) and four (MEOS-VSF) items per sub-scale were developed and validated. Study 1 used factor analysis of pre-existing MEOS item data to select items for the short forms and also compared the bivariate correlations of the MEOS, MEOS-SF and MEOS-VSF with personality and global trait EI. Study 2 examined the MEOS-SF and MEOS-VSF in two new samples (*N* = 394/226). The results from both studies showed that the short forms had good psychometric properties and associations similar to those of the full-length MEOS with personality, global trait EI, and other measures. The MEOS-SF and MEOS-VSF are hence suitable for use in contexts where a brief assessment of the full range of the domain of managing the emotions of others is required. The availability of short subscales assessing the manipulative facets of the MEOS is especially relevant to the emerging area of “dark side” trait EI research.

## Introduction

The Managing the Emotions of Others Scale (MEOS; Austin and O'Donnell, 2013) is a multidimensional scale which assesses interpersonal emotion management. Interpersonal emotional management is a facet of trait emotional intelligence (EI), which can be defined as “a distinct, compound trait located at the lower levels of personality hierarchies” which “comprehensively encompasses the emotion related facets of personality” (Petrides et al., 2007, p. 287). Managing another's emotions can be done a number of ways, which can be divided into two broad subtypes. One is a prosocial approach with the objective of helping the target, for example by attempting to reduce their negative emotions or distress. Alternatively, interpersonal emotion management can be used in a strategically manipulative manner to promote the objectives of the regulator rather than those of the target (e.g., Andrade and Ho, 2009; Netzer et al., 2015).

The MEOS was developed to provide broad coverage of the interpersonal emotional management domain. The test was developed using an item pool derived from a database of free-response descriptions of episodes of interpersonal emotion management; exploratory factor analysis was used to determine the number of underlying dimensions in the preliminary scale and reduce its length by selecting the highest-loading items and the resulting factor structure was confirmed in a second sample (Austin and O'Donnell, 2013). In addition to the English version of the scale, translations into Polish (Jankowski et al., 2016) and Mandarin (Saklofske et al., 2016) have been created.

The MEOS has four core subscales which assess aspects of actively managing the emotions of others. Improving another's mood is covered by the Enhance (e.g., offering help and reassurance) and Divert (diverting another's low mood, e.g., by use of humor) subscales. The Worsen subscale includes approaches to mood worsening such as criticism of and undermining the target, whilst the Inauthentic subscale includes the use of tactics such as flattery and sulking to influence another person's mood. Two additional subscales (Conceal, Poor Skills) cover concealing one's own emotions as an interpersonal emotion management tactic, and poor self-assessed ability to change another's mood. Enhance and Divert are related to the positive, generally prosocial aspects of trait EI, whilst Worsen and Inauthentic represent aspects of its “dark side” (Davis and Nichols, 2016). Collectively the four core subscales cover dispositional interpersonal emotion management tendencies that can have both positive and negative effects on relationships with others. Examination of correlations amongst the core MEOS subscales (e.g., Austin et al., 2014) shows that Enhance and Divert are strongly positively correlated, as are Worsen and Inauthentic, indicating that people who use one of the mood-improving strategies are likely to use the other as well and, conversely, those who use one of the less pleasant “dark” approaches to changing another's mood are likely to also use the other. Interestingly, the cross-correlations between the Enhance/Divert and Worsen/Inauthentic pairs are quite weak, indicating that people are generally able to use both prosocial and manipulative approaches to managing the moods of others (Austin and O'Donnell, 2013).

Studies of the associations of the MEOS with personality (Austin and O'Donnell, 2013; Austin et al., 2014; Austin and Vahle, 2016) have found theoretically-interpretable correlations. Enhance and Divert were found to be positively and Worsen and Inauthentic negatively correlated with Agreeableness, associations which are interpretable in terms of the relationships of Agreeableness with affiliation and positive interpersonal relationships (Jensen-Campbell and Graziano, 2001; Traupman et al., 2009). Worsen and Inauthentic show strong positive associations with the Dark Triad and strong negative associations with Honesty-Humility (H), as would be expected given that both the Dark Triad and low H are associated with interpersonal manipulation (e.g., Jakobwitz and Egan, 2006; Jonason et al., 2012; Lee et al., 2013). The MEOS has also been used to examine the mechanisms by which trait EI enables manipulative relational behaviors (Bacon and Regan, 2016).

Examination of the associations of the MEOS with trait EI has shown that Enhance and Divert are positively and Worsen and Inauthentic negatively correlated with global trait EI. At the trait EI facet level a more nuanced but interpretable correlation pattern emerges with, for example, all four core subscales being positively associated the Emotion Management subscale (assessing management of other's emotions) of the TEIQue (Petrides, 2009), but with the correlations with the TEIQue Empathy subscale mirroring the associations with total EI.

The MEOS comprises 58 items, a scale length which is not optimal for some study types, for example those employing multiple instruments or a repeat-measures-design, or in situations where participants are particularly likely to become bored or disengaged when completing a long scale. Given these considerations, the availability of shorter versions of the MEOS is desirable. Research designs requiring the use of short tests vary in the trade-off required between test length (with longer tests having generally better psychometric properties and construct domain coverage) and demands on participants (for example, experience sampling studies requiring completion of short surveys several times per day are more demanding than one-off surveys). To allow for the variation in these design issues, in this paper we develop two short versions of the MEOS containing six (MEOS-SF) and four (MEOS-VSF) items per subscale.

### Description of studies

The focus of the short form development was on the four core MEOS subscales together with Conceal. Earlier work on the MEOS has shown that the Poor Skills subscale scores have marginal internal reliability and that this subscale is not well-discriminated from global trait EI, with which has been found to correlate at around *r*, −0.60 (e.g., Austin and O'Donnell, 2013). Given the unsatisfactory psychometric properties of this subscale, this paper also incorporates a revision of the MEOS with Poor Skills omitted and focusses on creating short versions of the other five subscales. Study 1 used combined pre-existing data to develop two MEOS short forms using exploratory and confirmatory factor analysis, followed by examining the reliability and validity of scores of the new shorter versions. Study 2 re-examined the reliability and validity of the short form scores using two new samples and also extended results for the MEOS generally by examining its associations with several scales not included in earlier work.

## Study 1

### Method

This study used archival data from three previously-published papers (Austin and O'Donnell, 2013; Austin et al., 2014; Austin and Vahle, 2016).

#### Participants

Factor analysis was performed on the combined MEOS item data; full information on the component samples are provided in the publications cited above, and demographic information for the combined data used to develop the MEOS short forms is shown in Table 1. Each of the previous studies also included measures of personality and trait emotional intelligence (EI). The sizes of the subgroups used in the comparisons of correlations for the full-length and short MEOS scales with these measures are provided in the relevant table captions.

**Table 1:** Sample details for Study 1.

	***N***	**Percentage female**	**Mean (*SD*) age (years)**
Sample 1	2,005	73.6	22.86 (8.57)
Sample 2	341	71.6	22.51 (8.22)

*Samples 1 and 2 were randomly selected from the combined item-level MEOS data*.

#### Instruments

The scales listed below were used in the correlation comparisons. Descriptive statistics for the scale scores and scale internal reliabilities are reported in the previously-published papers using these datasets (Austin and O'Donnell, 2013; Austin et al., 2014; Austin and Vahle, 2016).

##### Mini-markers (Saucier, 1994)

This set of 40 trait-descriptive adjectives provides scores on the Big Five dimensions of Neuroticism (N), Extraversion (E), Openness (O), Agreeableness (A), and Conscientiousness (C). Responses are on a five-point scale indicating how accurately the adjective describes the respondent, with end points very inaccurately, very accurately.

##### HEXACO-60 (Ashton and Lee, 2009)

This 60-item scale assesses the personality dimensions of Honesty-Humility (H), Emotionality (E), Extraversion (X), Agreeableness (A), Conscientiousness (C), and Openness (O). Responses are on a five-point scale with end points strongly disagree, strongly agree.

##### Mach IV (Christie and Geis, 1970)

This 20-item scale assesses Machiavellianism, with responses on a five-point scale with end points strongly disagree, strongly agree.

##### NPI-16 (Ames et al., 2006)

This scale has 16 forced-choice items assessing grandiose narcissism.

##### Hypersensitive narcissism scale (Hendin and Cheek, 1997)

This 10-item scale, assesses vulnerable narcissism, with responses on a five-point scale with end points strongly disagree, strongly agree.

##### Levenson self-report psychopathy scale (Levenson et al., 1995)

This scale assesses primary (16 items) and secondary (10 items) psychopathy in general population samples, with responses on a five-point scale with end points strongly disagree, strongly agree.

##### Trait EI

Results are reported for total scores on the 144-item TEIQue (Petrides, 2009) and the 30-item TEIQue-SF (Petrides and Furnham, 2006). These measures have a 7-point response scale with end points completely disagree, completely agree.

#### Data analysis

The combined item-level dataset was used to produce versions of the four core MEOS sub-scales and Conceal with six (MEOS Short Form: MEOS-SF) and four (MEOS Very Short Form: MEOS-VSF) items per subscale. These subscale lengths were selected to allow considerable shortening of the MEOS without excessive decrements in subscale score reliability or validity (Credé et al., 2012). Initial item selection was based on those items with highest loadings on their respective factors in an exploratory factor analysis, but a small number of items were substituted with lower-loading items to avoid having too many items with similar wordings and to maximize the construct breadth of the short forms. The exploratory factor analysis was repeated on the 30 items of the MEOS-SF and the 20 items of the MEOS-VSF to re-check that each item still had its principal loading on its designated factor. A confirmatory factor analysis was also performed on a subset of the combined data not included in the exploratory analysis. The correlations of the full MEOS, the MEOS-SF and the MEOS-VSF with personality and trait EI were compared to examine how similar the validity coefficients for scores on the three MEOS versions were, and the internal reliabilities of the MEOS-SF and MEOS-VSF subscale scores were examined.

### Results

#### Exploratory factor analysis and item selection

A subset comprising a randomly selected 85% of the item-level data (Sample 1) was used for the exploratory factor analysis (EFA) stage of scale development, with the remaining 15% (Sample 2) being used for confirmatory factor analysis (CFA). Information about the two samples is shown in Table 1. EFA was performed on Sample 1 using principal axis factoring with direct oblimin rotation.

The standard approach in short-form test development when the factor structure of the full-length test has been previously established is to use this structure of the starting point for item selection (e.g., Donnellan et al., 2006; Van der Zee et al., 2013). In the case of the MEOS, both exploratory (Austin and O'Donnell, 2013, Studies 2 and 3) and confirmatory factor analysis (Austin and O'Donnell, Study 3) have previously established the six-factor structure described in the section Introduction. The scree criterion and parallel analysis methods for factor number determination were however examined in the current data to verify that there was no major inconsistency with the previously-established factor structure. The scree criterion indicated the extraction of six factors, whilst parallel analysis marginally indicated seven factors, with the seventh sample and randomly-generated seventh eigenvalues being numerically very similar. Examination of the seven-factor solution showed that this differed from the six-factor one in splitting the Inauthentic factor into two sub-factors. To preserve the consistency of the short forms with the established MEOS factor structure, retaining a unitary Inauthentic factor, the item content of the short forms was derived from the six-factor solution, which showed good simple structure, with the factors explaining 41% of the variance.

The Poor Skills factor was not examined further and was excluded from subsequent analyses. For the other five factors, the six highest-loading items on each were reviewed for content. For Enhance there were four items which referred to approaches to helping an anxious person. As the focus of the MEOS is on approaches to managing the emotions of others rather than the specific emotional state of the target, in order to reduce the number of anxiety items, the lowest-loading item of this type (item 50: *If someone is feeling anxious, I try to offer practical help*) was replaced with the next highest-loading item (item 57: *If someone has a problem I offer to help if they need it*). For Worsen there were two similarly-worded items referring to the use of anger to get others to act as desired. The lower-loading of these (item 16: *I use displays of anger to motivate others*) was replaced with the next highest-loading item (item 20: *If I don't like someone's behavior I make negative comments in order to make them feel bad*). For Inauthentic three high-loading items referred to sulking. The lowest-loading of these (item 9: *If someone says or does something I don't like, I sometimes sulk*) was replaced with the next highest-loading item (item 2: If I want someone to do something for me, I am especially nice to them before asking). An EFA of the 30 items selected for the MEOS-SF using principal axis factoring with direct oblimin rotation and specifying five factors was performed. The factor pattern matrix showed good simple structure. Compared to the initial analysis using all the MEOS items there were some changes in the rank order of loadings within each factor. Each item had its principal loading on its subscale, with all but two of these being above 0.4 (two values of 0.36 for an Inauthentic and a Conceal item). The four highest-loading items for each factor from this analysis were selected for the MEOS-VSF and the EFA was repeated with these items, again resulting in good simple structure and only one principal loading below 0.4 (0.36 for an Inauthentic item). Tables 2, 3 show the factor structures of the MEOS-SF and MEOS-VSF, Table 4 shows the factor score correlations, which showed the same structure as for the full-length MEOS, with strong correlations between the pairs Enhance/Divert and Worsen/Inauthentic with the remaining correlations low. Table 5 shows the item wordings for the two short forms.

**Table 2:** Exploratory factor analysis of the MEOS-SF.

	**Enhance**	**Inauthentic**	**Conceal**	**Worsen**	**Divert**
Enhance 3	**0.74**	0.04	0.01	−0.05	−0.04
Enhance 1	**0.74**	−0.01	−0.02	0.01	0.02
Enhance 2	**0.73**	−0.05	0.00	0.03	−0.02
Enhance 4	**0.69**	−0.04	0.01	0.05	0.10
Enhance 5	**0.63**	0.06	0.01	0.02	0.01
Enhance 6	**0.58**	−0.06	0.02	−0.03	0.07
Inauthentic 1	0.01	**0.81**	−0.07	−0.08	−0.11
Inauthentic 3	0.04	**0.67**	−0.12	0.10	−0.11
Inauthentic 2	−0.01	**0.65**	−0.04	0.11	−0.07
Inauthentic 6	−0.04	**0.46**	0.06	0.00	0.17
Inauthentic 5	−0.06	**0.45**	0.04	0.19	0.03
Inauthentic 4	−0.05	*0.36*	0.11	0.14	0.20
Conceal 1	0.08	0.01	**0.75**	0.02	−0.09
Conceal 2	0.04	−0.08	**0.71**	0.07	−0.01
Conceal 3	−0.01	0.03	**0.67**	−0.12	0.00
Conceal 4	0.02	−0.01	**0.66**	−0.10	−0.01
Conceal 5	−0.10	−0.13	**0.60**	0.13	−0.03
Conceal 6	0.03	0.23	*0.36*	−0.02	0.12
Worsen 2	−0.05	0.02	0.03	**0.68**	−0.02
Worsen 5	0.03	−0.05	0.03	**0.63**	0.00
Worsen 3	0.07	0.03	−0.02	**0.62**	0.02
Worsen 6	0.07	0.10	−0.07	**0.62**	−0.07
Worsen 1	−0.05	0.05	−0.02	**0.61**	−0.07
Worsen 4	−0.12	0.05	0.01	**0.61**	0.10
Divert 1	−0.01	−0.01	−0.05	−0.02	**0.71**
Divert 3	−0.01	−0.04	0.00	0.09	**0.70**
Divert 2	0.02	−0.02	−0.03	−0.04	**0.66**
Divert 5	0.06	0.00	0.00	−0.01	**0.51**
Divert 4	0.29	0.00	0.01	−0.07	**0.46**
Divert 6	0.13	0.05	0.01	−0.06	**0.46**

*The factors accounted for 43% of the variance. Pattern matrix elements are shown. Loadings ≥ 0.4 are shown in bold, below 0.4 but > 0.3 in italic. Items are numbered in order of loading size in the EFA of the full MEOS. See Table 5 for item wordings*.

**Table 3:** Exploratory factor analysis of the MEOS-VSF.

	**Enhance**	**Inauthentic**	**Conceal**	**Worsen**	**Divert**
Enhance 3	**0.74**	0.03	0.02	−0.02	−0.04
Enhance 1	**0.73**	0.01	−0.01	−0.01	0.03
Enhance 2	**0.73**	−0.03	0.00	0.01	−0.02
Enhance 4	**0.69**	−0.02	0.01	0.02	0.10
Inauthentic 1	0.00	**0.91**	−0.01	−0.13	−0.06
Inauthentic 3	0.03	**0.66**	−0.09	0.12	−0.07
Inauthentic 2	−0.03	**0.64**	−0.01	0.10	−0.04
Inauthentic 6	−0.02	*0.36*	0.06	0.05	0.18
Conceal 1	0.05	0.02	**0.75**	0.05	−0.06
Conceal 3	−0.04	0.04	**0.72**	−0.08	0.01
Conceal 4	0.00	−0.01	**0.70**	−0.06	−0.01
Conceal 2	0.02	−0.06	**0.63**	0.07	0.02
Worsen 6	0.05	0.06	−0.07	**0.68**	−0.06
Worsen 2	−0.09	0.01	0.03	**0.67**	0.00
Worsen 3	0.03	0.02	−0.03	**0.60**	0.03
Worsen 5	−0.02	−0.02	0.04	**0.57**	0.01
Divert 3	−0.05	−0.04	0.01	0.09	**0.78**
Divert 1	0.05	0.02	−0.03	−0.06	**0.63**
Divert 2	0.04	0.01	−0.02	−0.07	**0.62**
Divert 5	0.06	−0.01	0.02	0.01	**0.53**

*The factors accounted for 47% of the variance. Pattern matrix elements are shown. Loadings ≥ 0.4 are shown in bold, below 0.4 but > 0.3 in italic. Items are numbered in order of loading size in the EFA of the full MEOS. See Table 5 for item wordings*.

**Table 4:** Factor score bivariate correlations for the MEOS-SF and MEOS-VSF.

	**Enhance**	**Divert**	**Worsen**	**Inauthentic**
**MEOS-SF**				
Divert	**0.53**			
Worsen	−0.21	−0.07		
Inauthentic	−0.07	0.08	**0.47**	
Conceal	0.06	0.14	0.00	0.00
**MEOS-VSF**				
Divert	**0.43**			
Worsen	−0.16	−0.02		
Inauthentic	−0.07	0.05	**0.39**	
Conceal	0.08	0.11	−0.04	−0.10

*N = 2,005; substantive correlations shown in bold*.

**Table 5:** Items selected for the MEOS-SF and MEOS-VSF.

**MEOS item**	**Item numbers used in Tables 2, 3**	
**ENHANCE**		
43	Enhance 1	*If someone is feeling anxious, I try to calm them down by talking with them*.
38	Enhance 2	*When someone is anxious about a problem, I try to help them work out a solution*.
28	Enhance 3	*If someone is anxious, I try to reassure them*.
35	Enhance 4	*When someone is under stress I try to boost their confidence in their ability to cope*.
31	Enhance 5	When someone is unhappy, I show that I understand how they are feeling.
57	Enhance 6	If someone has a problem I offer to help if they need it.
**DIVERT**		
46	Divert 1	*If someone is angry, I try to divert their mood by being cheerful*.
53	Divert 2	*When someone is in a low mood I behave in a happy and cheerful way to make them feel better*.
33	Divert 3	*When someone is in a bad mood I try to divert them by telling jokes or funny stories*.
22	Divert 4	When someone is unhappy I try to cheer them by talking about something positive.
3	Divert 5	*I sometimes use humor to try to lift another person's mood*.
45	Divert 6	If someone is being awkward, I try to defuse the situation by being cheerful and pleasant.
**WORSEN**		
21	Worsen 1	I use anger to get others to do things that I want them to do.
19	Worsen 2	*I sometimes put someone down in public to make them feel bad*.
32	Worsen 3	*I know how to make someone feel ashamed about something that they have done in order to stop them from doing it again*.
47	Worsen 4	I can make someone feel anxious so that they will act in a particular way.
29	Worsen 5	*I use criticism to make others feel that they should work harder*.
20	Worsen 6	*If I don't like someone's behavior I make negative comments in order to make them feel bad*.
**INAUTHENTIC**		
4	Inauthentic 1	*I sometimes sulk to make someone feel guilty*.
44	Inauthentic 2	*I sometimes sulk to get someone to change their behavior*.
5	Inauthentic 3	*If someone's behavior has caused me distress, I try to make them feel guilty about it*.
37	Inauthentic 4	I sometimes use flattery to gain or keep someone's good opinion.
12	Inauthentic 5	If I want someone to do something for me, I try to elicit sympathy from them.
2	Inauthentic 6	*If I want someone to do something for me, I am especially nice to them before asking*.
**CONCEAL**		
30	Conceal 1	*I often conceal feelings of anger and distress from others*.
36	Conceal 2	*I hide my feelings so others won't worry about me*.
8	Conceal 3	*When someone has made me upset or angry, I often conceal my feelings*.
18	Conceal 4	*When someone has made me upset or angry, I tend to downplay my feelings*.
23	Conceal 5	I don't believe in telling others about my problems – I keep them to myself.
26	Conceal 6	If someone tries to make me feel better when I am feeling low, I pretend to feel happier to please that person.

*All 30 items are used in the MEOS-SF. The 24 items of the MEOS-VSF are shown in italic*.

#### Confirmatory factor analysis

CFA of the MEOS-SF and MEOS-VSF was performed on Sample 2 using EQS 6.3. Fit indices for a simple structure model with correlated factors for each scale are shown in Table 6. All fit indices other than the comparative fit index for the MEOS-SF (0.88, below the acceptable fit cut-off of 0.90) fell in the acceptable fit range (e.g., Schweizer, 2010). It would be possible to improve model fit for both scales by model modifications (adding cross-loadings and/or correlated error terms) but this risks creating a model which fails to generalize to new samples (MacCallum et al., 1992), so modified models are not presented here.

**Table 6:** CFA model fit.

	**χ^2^(*df*)**	**χ^2^/*df***	**CFI**	**SRMR**	**RMSEA (90% CI)**
MEOS-SF	834.73 (395)	2.11	0.88	0.071	0.057 (0.052,0.063)
MEOS-VSF	323.62 (160)	2.02	0.93	0.057	0.055 (0.046,0.063)

*CFI, comparative fit index; SRMR, standardized root mean square residual; RMSEA, root mean square error of approximation*.

#### Internal reliability and validity

Table 7 shows internal reliabilities, using the omega coefficient (Dunn et al., 2014; Trizano-Hermosilla and Alvarado, 2016) for the MEOS-SF and MEOS-VSF scores; all subscale scores showed satisfactory internal reliability. Comparisons of bivariate correlations of the MEOS, MEOS-SF, and MEOS-VSF with personality traits (Five–Factor and HEXACO), the Dark Triad and trait EI are shown in Tables 8–10. These results show that the substantive correlations with other scales were of comparable size for the full-length MEOS and its short forms.

**Table 7:** Internal reliabilities (omega coefficients) for Sample 1.

	**MEOS-SF**	**MEOS-VSF**
Enhance	0.87	0.84
Divert	0.82	0.79
Worsen	0.83	0.77
Inauthentic	0.79	0.78
Conceal	0.81	0.79

*MEOS-SF, short form with six items per subscale; MEOS-VSF, very short form with four items per subscale*.

**Table 8:** Bivariate correlations of the MEOS, MEOS-SF, and MEOS-VSF with personality and trait EI using data from Austin et al. (2014) Study 1.

	**Enh**	**Enh-SF**	**Enh-VSF**	**Div**	**Div-SF**	**Div-VSF**	**Wor**	**Wor-SF**	**Wor-VSF**	**Inau**	**Inau-SF**	**Inau-VSF**	**Conc**	**Conc-SF**	**Conc-VSF**
NPI	−**0.16**	−**0.15**	−0.12	−0.04	−0.03	−0.03	**0.44**	**0.41**	**0.39**	**0.31**	**0.26**	**0.22**	−**0.17**	−0.13	−**0.18**
HSN	−**0.14**	−**0.16**	−**0.13**	−0.11	−0.10	−0.07	**0.38**	**0.36**	**0.33**	**0.50**	**0.45**	**0.40**	0.00	0.04	0.00
P1	−**0.44**	−**0.44**	−**0.42**	−**0.21**	−**0.21**	−**0.16**	**0.56**	**0.53**	**0.47**	**0.44**	**0.38**	**0.33**	−0.07	−0.02	−0.08
P2	−**0.33**	−**0.34**	−**0.33**	−**0.23**	−**0.23**	−0.15	**0.48**	**0.44**	**0.39**	**0.38**	**0.34**	**0.35**	−0.10	−0.04	−0.09
Mach	−**0.30**	−**0.28**	−**0.26**	−0.13	−0.12	−0.08	**0.42**	**0.38**	**0.34**	**0.37**	**0.30**	**0.24**	0.03	0.07	0.00
EI	**0.39**	**0.40**	**0.38**	**0.31**	**0.33**	**0.27**	−**0.24**	−**0.23**	−**0.19**	−**0.26**	−**0.24**	−**0.25**	−0.11	−0.13	−0.10
N	−**0.15**	−**0.13**	−0.11	−**0.16**	−**0.15**	−**0.14**	**0.34**	**0.30**	**0.28**	**0.42**	**0.37**	**0.36**	−**0.15**	−0.12	−**0.14**
E	**0.14**	0.13	0.12	**0.27**	**0.26**	**0.23**	0.04	0.02	0.01	−0.05	−0.04	−0.05	−**0.25**	−**0.24**	−**0.21**
O	**0.23**	**0.25**	**0.22**	**0.17**	**0.16**	**0.16**	0.02	0.01	0.04	0.01	0.00	−0.02	−0.04	−0.03	−0.05
A	**0.54**	**0.53**	**0.48**	**0.36**	**0.36**	**0.29**	−**0.53**	−**0.45**	−**0.41**	−**0.35**	−**0.33**	−**0.33**	**0.15**	0.10	**0.16**
C	**0.29**	**0.28**	**0.27**	**0.27**	**0.27**	**0.23**	−**0.20**	−**0.19**	−**0.16**	−**0.15**	−0.12	−**0.15**	0.03	0.01	0.01

*Enh, Enhance; Div, Divert; Wor, Worsen; Inau, Inauthentic; Conc, Conceal; NPI, Narcissistic Personality Inventory; HSN, Hypersensitive Narcissism Scale; P1, P2, primary, secondary psychopathy; Mach, Machiavellianism; N, E, O, A, C, five-factor model Neuroticism, Extraversion, Openness, Agreeableness, Conscientiousness. N range 349–370. Correlations with p ≤ 0.01 shown in bold*.

**Table 9:** Bivariate correlations of the MEOS, MEOS-SF, and MEOS-VSF with personality and trait EI using data from Austin et al. (2014) Study 2.

	**Enh**	**Enh-SF**	**Enh-VSF**	**Div**	**Div-SF**	**Div-VSF**	**Wor**	**Wor-SF**	**Wor-VSF**	**Inau**	**Inau-SF**	**Inau-VSF**	**Conc**	**Conc-SF**	**Conc-VSF**
NPI	−**0.13**	−**0.16**	−**0.16**	−0.10	−0.07	−0.08	**0.37**	**0.37**	**0.32**	**0.25**	**0.18**	0.13	−0.12	−0.09	−0.10
HSN	−**0.17**	−**0.17**	−**0.17**	−**0.17**	−**0.17**	−**0.16**	**0.40**	**0.35**	**0.31**	**0.51**	**0.46**	**0.44**	**0.14**	**0.19**	**0.15**
P1	−**0.40**	−**0.40**	−**0.40**	−**0.20**	−**0.20**	−**0.16**	**0.56**	**0.52**	**0.47**	**0.44**	**0.36**	**0.32**	−0.04	−0.02	−0.06
P2	−**0.29**	−**0.30**	−**0.28**	−**0.14**	−**0.14**	−0.10	**0.40**	**0.36**	**0.32**	**0.34**	**0.31**	**0.32**	0.04	0.07	0.01
Mach	−**0.35**	−**0.31**	−**0.31**	−**0.25**	−**0.24**	−**0.20**	**0.45**	**0.40**	**0.37**	**0.39**	**0.33**	**0.30**	**0.14**	**0.16**	0.08
EI	**0.40**	**0.40**	**0.37**	**0.34**	**0.35**	**0.31**	−0.13	−0.11	−0.07	−**0.22**	−**0.23**	−**0.28**	−**0.20**	−**0.20**	−0.13
N	−0.07	−0.08	−0.07	−**0.14**	−**0.14**	−**0.16**	**0.21**	**0.15**	**0.13**	**0.45**	**0.41**	**0.44**	−0.10	−0.08	−**0.14**
E	**0.14**	0.11	0.10	**0.29**	**0.29**	**0.28**	0.09	0.06	0.05	0.09	0.08	0.04	−**0.31**	−**0.31**	−**0.30**
O	**0.14**	**0.15**	**0.17**	−0.01	−0.01	−0.04	0.04	0.02	0.03	0.06	0.03	0.00	−0.01	0.02	0.05
A	**0.52**	**0.49**	**0.45**	**0.38**	**0.35**	**0.30**	−**0.49**	−**0.49**	−**0.46**	−**0.24**	−**0.20**	−**0.21**	0.04	0.02	0.07
C	**0.21**	**0.18**	**0.16**	0.07	0.07	0.04	−0.10	−0.07	−0.08	−**0.14**	−**0.14**	−**0.14**	−0.12	−0.10	−0.07

*Enh, Enhance; Div, Divert; Wor, Worsen; Inau, Inauthentic; Conc, Conceal; NPI, Narcissistic Personality Inventory; HSN, Hypersensitive Narcissism Scale; P1, P2, primary, secondary psychopathy; Mach, Machiavellianism; N, E, O, A, C, five-factor model Neuroticism, Extraversion, Openness, Agreeableness, Conscientiousness. N range 362–387. Correlations with p ≤ 0.01 shown in bold*.

**Table 10:** Bivariate correlations of the MEOS, MEOS-SF, and MEOS-VSF with personality and trait EI using data from Austin and Vahle (2016).

	**Enh**	**Enh-SF**	**Enh-VSF**	**Div**	**Div-SF**	**Div-VSF**	**Wor**	**Wor-SF**	**Wor-VSF**	**Inau**	**Inau-SF**	**Inau-VSF**	**Conc**	**Conc-SF**	**Conc-VSF**
H	**0.20**	**0.21**	**0.18**	−0.01	−0.02	−0.03	−**0.40**	−**0.35**	−**0.31**	−**0.55**	−**0.50**	−**0.41**	0.05	0.03	0.06
E	**0.23**	**0.22**	**0.21**	0.12	0.11	0.09	−0.13	−**0.13**	−0.11	**0.17**	**0.16**	**0.18**	−0.11	−0.12	−0.13
X	**0.29**	**0.23**	**0.23**	**0.34**	**0.33**	**0.30**	0.00	0.02	0.02	−0.06	−0.07	−0.13	−**0.18**	−**0.15**	−**0.13**
A	**0.27**	**0.25**	**0.22**	0.12	0.11	0.08	−**0.42**	−**0.38**	−**0.36**	−**0.28**	−**0.25**	−**0.28**	**0.29**	**0.25**	**0.31**
C	**0.20**	**0.21**	**0.21**	0.01	0.01	−0.02	−**0.26**	−**0.20**	−**0.20**	−**0.29**	−**0.28**	−**0.29**	−0.01	−0.02	0.02
O	**0.17**	**0.20**	**0.20**	0.04	0.06	0.03	−0.07	−0.08	−0.08	−0.05	−0.04	−0.06	0.01	−0.01	0.03
EI	**0.44**	**0.38**	**0.37**	**0.32**	**0.31**	**0.27**	−**0.22**	−**0.18**	−**0.18**	−**0.27**	−**0.27**	−**0.31**	−0.05	−0.05	−0.02

*Enh, Enhance; Div, Divert; Wor, Worsen; Inau, Inauthentic; Conc, Conceal; H, E, X A, C, O, HEXACO model Honesty-Humility, Emotionality, Extraversion, Agreeableness, Conscientiousness, Openness; N range 390–398. Correlations with p ≤ 0.01 shown in bold*.

### Discussion

The development of the MEOS-SF and MEOS-VSF from data available from previous studies resulted in the subscale scores displaying good internal reliability, whilst the factor structure of both short forms was the same as that found for the full-length MEOS. CFA fit was satisfactory rather than good, but the context for this result is that CFA models for multidimensional scales at both the facet and item level often fail to achieve good fit (e.g., Aluja et al., 2004). For a direct comparison for a similar length scale, see Donnellan et al. (2006), who reported similar fit indices to those for the MEOS-SF for a simple structure model for the 25-item Mini-IPIP scale. In addition, the correlations of the MEOS-SF and MEOS-VSF with personality and trait EI were similar to those for the full-length MEOS, indicating similar validity for the subscale scores for the full-length MEOS and its short forms. As it is important to verify the reliability and validity of the MEOS short form scores using independent data, a second study was undertaken, which also included several measures whose associations with the MEOS have not previously been examined.

## Study 2

### Method

#### Ethics statement

Two new datasets were obtained for this study. Ethical approval for the study was obtained from the relevant university ethics committees: the Ethics Committee of the Psychology Department, University of Western Ontario for Sample 3 and the Ethics Committee of the School of Philosophy, Psychology and Language Sciences, University of Edinburgh for Sample 4.

#### Participants

Sample 3 comprised 394 students at the University of Western Ontario who participated for course credit. The sample comprised 78 males, 316 females and were mostly (98%) in the age group 17–22 years. A subset of 116 students completed a retest survey, with a mean test-retest interval of 34 days. Sample 4 comprised 226 students at the University of Edinburgh who participated for course credit. The sample comprised 54 males, 172 females. The mean age was 19.3 years, standard deviation 3.1 years. A subset of 36 students completed a retest survey, with a mean test-retest interval of 32 days.

#### Instruments

In addition to the MEOS, the survey contained the scales listed below. Internal reliabilities for all scale scores are presented in Table 11.

**Table 11:** Descriptive statistics, internal and test-retest reliabilities for samples 3 and 4.

	**Sample 3**			**Sample 4**		
	**Mean (*SD*)**	**Omega**	**Test-Retest**	**Mean (*SD*)**	**Omega**	**Test-Retest**
Enhance	60.03(8.38)	0.91	0.65	63.32(6.81)	0.89	0.88
Divert	27.43(4.10)	0.73	0.57	27.41(4.06)	0.78	0.80
Worsen	30.80(9.53)	0.90	0.70	25.76(7.92)	0.87	0.78
Inauthentic	32.66(7.03)	0.83	0.74	31.16(6.95)	0.79	0.82
Conceal	23.26(4.91)	0.80	0.69	24.37(5.30)	0.82	0.84
Enhance-SF	23.83(3.59)	0.82	0.55	25.45(3.05)	0.84	0.82
Divert-SF	23.70(3.70)	0.72	0.60	23.65(3.63)	0.77	0.82
Worsen-SF	14.20(4.67)	0.80	0.60	11.54(3.92)	0.79	0.82
Inauthentic-SF	18.05(4.18)	0.78	0.69	17.23(4.09)	0.72	0.75
Conceal-SF	20.03(4.50)	0.79	0.63	20.85(4.74)	0.82	0.83
Enhance-VSF	15.83(2.55)	0.80	0.50	16.91(2.22)	0.82	0.78
Divert-VSF	15.79(2.71)	0.66	0.63	15.53(2.62)	0.70	0.78
Worsen-VSF	9.79(3.26)	0.72	0.55	8.21(2.89)	0.72	0.83
Inauthentic-VSF	11.77(3.02)	0.77	0.65	11.45(3.09)	0.74	0.68
Conceal-VSF	13.64(3.29)	0.79	0.61	14.46(3.43)	0.80	0.72
H	31.58(6.16)	0.73		33.32(6.42)	0.73	
E	34.21(6.34)	0.75		34.57(6.45)	0.75	
X	32.59(6.86)	0.83		31.60(7.56)	0.85	
A	31.33(6.50)	0.79		33.17(6.87)	0.82	
C	35.67(6.45)	0.81		33.86(6.86)	0.82	
O	32.12(6.76)	0.78		34.96(6.64)	0.76	
SEA	20.08(5.06)	0.89		19.22(4.85)	0.84	
OEA	21.56(4.28)	0.84		21.32(4.19)	0.85	
UOE	21.20(4.78)	0.84		20.05(4.52)	0.77	
ROE	19.13(5.76)	0.90		18.50(4.72)	0.82	
TEIQue-SF total score	141.78(21.43)	0.87				
Emotion Management				41.21(7.24)	0.73	
Relationship Skills				49.70(6.86)	0.69	
Social Competence				50.26(10.32)	0.85	
Empathy				47.31(7.48)	0.80	
Extrinsic Affect Improving	23.45(3.56)	0.86	0.64	22.40(4.44)	0.84	
Extrinsic Affect Worsening	5.83(2.49)	0.80	0.47	4.55(1.86)	0.75	
Intrinsic Affect Improving	20.08(4.92)	0.84	0.61	19.52(4.85)	0.79	
Intrinsic Affect Worsening	9.48(4.03)	0.89	0.62	8.99(3.70)	0.85	
PA	34.21(7.60)	0.89	0.70	32.02(7.09)	0.86	
NA	23.41(7.75)	0.87	0.55	26.00(7.56)	0.85	
Life Satisfaction	24.39(6.59)	0.89	0.76	23.42(6.55)	0.86	

*H, E, X, A, C, O, Honesty-Humility, Emotionality, Extraversion, Agreeableness, Conscientiousness, Openness; SEA, self-emotional appraisal; OEA, others' emotional appraisal; UOE, use of emotion; ROE, regulation of emotion in self; PA, positive affect; NA, negative affect*.

##### HEXACO-60 (Ashton and Lee, 2009)

This 60-item scale assesses the personality dimensions of Honesty-Humility (H), Emotionality (E), Extraversion (X), Agreeableness (A), Conscientiousness (C), and Openness (O). Responses are on a five-point scale with endpoints strongly disagree, strongly agree.

##### Trait EI

Sample 3 participants completed the 30-item TEIQue-SF (Petrides and Furnham, 2006). Sample 4 participants completed selected subscales of the full TEIQue (Petrides, 2009) which fall within the interpersonal trait EI domain (Emotion Management, Relationship Skills, Social Competence and Empathy). These scales have a 7-point response scale with endpoints disagree completely, agree completely. All participants also completed the Wong and Law Emotional Intelligence Scale (WLEIS; Wong and Law, 2002). This 16-item scale has subscales Self-emotion appraisal (SEA), Others' emotion appraisal (OEA), Use of emotion (UOE) and Regulation of emotion (ROE). The test has a seven-point response scale with endpoints completely disagree, completely agree.

##### Emotion regulation of others and self-scale (EROS; Niven et al., 2011)

This 19-item scale has two subscales relating to regulation of own emotions (Intrinsic Affect Improving and Worsening) and two relating to regulation of the emotions of others (Extrinsic Affect Improving and Worsening). Responses are on a five-point scale with endpoints not at all, a great deal.

##### Positive and negative affect schedule (PANAS; Watson et al., 1988)

The 20-item (10 items for positive affect, PA and 10 for negative effect, NA) version of this scale was used. For each emotion listed participants responded on a five-point scale from very slightly or not at all to extremely, indicating the extent to which they had experienced that emotion during the past week.

##### Life satisfaction (Diener et al., 1985)

The five-item Satisfaction With Life Scale (SWLS) has responses on a 7-point scale with endpoints strongly disagree, strongly agree.

For Sample 3 the retest survey included the MEOS, EROS, PANAS and SWLS, whilst only the MEOS was included in the retest for Sample 4.

#### Procedure

All surveys were completed online. Participants were recruited via their respective undergraduate subject pools. On completion of the initial survey participants could choose to provide a contact email if interested in completing a follow-up survey. Participants who provided an email were subsequently contacted with a link allowing access to the retest survey.

#### Data analysis

Reliability (internal and test-retest) of the MEOS-SF and MEOS-VSF scores were examined and the correlations of the full MEOS and the short forms with the other study measures were examined. As the associations of the MEOS with the EROS, WLEIS, PANAS, and SWLS have not been reported previously, these are described in a separate section, including results which cross-validate the MEOS with the Extrinsic subscales of the EROS.

### Results

#### Reliability and validity

Table 11 shows descriptive statistics, and the internal reliabilities and test-retest reliabilities of scores on the MEOS versions and other study measures.

Apart from MEOS-VSF Divert in Sample 3, all short MEOS subscale scores had internal reliabilities above 0.7. Test-retest reliabilities were good for subscale scores of all versions of the MEOS in Sample 4, with only the MEOS-VSF Inauthentic score test-retest reliability falling below 0.7; other values were comparable to the range 0.71–0.83 previously reported for scores on the full-length MEOS (Austin and O'Donnell, 2013). Test-retest reliabilities of scores on the MEOS versions in Sample 3 were lower and were also similarly low for EROS subscale scores.

Comparisons of bivariate correlations of the MEOS, MEOS-SF and MEOS-VSF with personality, trait EI, EROS, PA, NA and life satisfaction are shown in the Tables 12, 13. As in Study 1, these results show that the substantive correlations with other scales were of comparable size for the full-length MEOS and its short forms.

**Table 12:** Bivariate correlation comparisons for Sample 3.

	**Enh**	**Enh-SF**	**Enh-VSF**	**Div**	**Div-SF**	**Div-VSF**	**Wor**	**Wor-SF**	**Wor-VSF**	**Inau**	**Inau-SF**	**Inau-VSF**	**Conc**	**Conc-SF**	**Conc-VSF**
H	**0.26**	**0.24**	**0.22**	0.08	0.05	0.05	−**0.48**	−**0.44**	−**0.42**	−**0.54**	−**0.46**	−**0.36**	0.06	0.05	0.08
E	**0.18**	**0.18**	**0.15**	**0.18**	**0.17**	**0.17**	−0.12	−0.12	−0.13	**0.16**	**0.18**	**0.21**	−**0.18**	−**0.17**	−**0.16**
X	**0.28**	**0.27**	**0.26**	**0.33**	**0.32**	**0.28**	−0.08	−0.05	−0.03	−0.11	−0.09	−0.12	−**0.21**	−**0.22**	−**0.20**
A	**0.26**	**0.23**	**0.19**	**0.17**	**0.15**	0.12	−**0.42**	−**0.38**	−**0.35**	−**0.36**	−**0.33**	−**0.33**	**0.28**	**0.23**	**0.27**
C	**0.25**	**0.25**	**0.24**	**0.17**	**0.16**	**0.14**	−**0.29**	−**0.27**	−**0.25**	−**0.26**	−**0.20**	−**0.17**	0.01	−0.01	0.01
O	**0.22**	**0.23**	**0.25**	**0.16**	**0.13**	0.08	−**0.14**	−**0.13**	−0.12	−**0.13**	−**0.15**	−**0.19**	0.12	0.12	0.09
SEA	**0.22**	**0.19**	**0.16**	**0.16**	**0.15**	0.12	−0.09	−0.06	−0.02	−0.12	−0.06	−0.03	−0.10	−0.10	−0.09
OEA	**0.47**	**0.46**	**0.41**	**0.40**	**0.37**	**0.36**	−**0.24**	−**0.24**	−**0.22**	−**0.18**	−**0.14**	−**0.14**	0.07	0.06	0.08
UOE	**0.29**	**0.28**	**0.27**	**0.29**	**0.29**	**0.27**	−**0.15**	−0.13	−0.09	−**0.14**	−0.12	−**0.13**	−0.05	−0.06	−0.04
ROE	**0.15**	**0.14**	0.12	**0.17**	**0.17**	**0.16**	−**0.18**	−0.13	−0.10	−**0.20**	−**0.16**	−**0.17**	**0.23**	**0.20**	**0.24**
EI	**0.35**	**0.33**	**0.31**	**0.30**	**0.29**	**0.24**	−**0.22**	−**0.18**	−**0.13**	−**0.27**	−**0.26**	−**0.28**	−**0.18**	−**0.20**	−**0.15**
Extr Aff Imp	**0.58**	**0.56**	**0.52**	**0.53**	**0.51**	**0.48**	−**0.24**	−**0.23**	−**0.23**	−**0.13**	−0.09	−0.11	0.10	0.09	0.07
Extr Aff Wors	−**0.27**	−**0.27**	−**0.24**	−**0.14**	−**0.15**	−**0.13**	**0.64**	**0.58**	**0.55**	**0.46**	**0.43**	**0.41**	−**0.15**	−0.11	−**0.15**
Intr Aff Imp	**0.23**	**0.20**	**0.18**	**0.32**	**0.31**	**0.29**	0.01	0.00	0.00	0.07	0.10	0.07	−0.11	−0.11	−0.11
Intr Aff Wors	−0.11	−0.10	−0.09	−0.08	−0.08	−0.05	**0.28**	**0.26**	**0.21**	**0.26**	**0.23**	**0.21**	**0.13**	**0.15**	0.10
PA	**0.35**	**0.29**	**0.28**	**0.33**	**0.30**	**0.26**	−0.06	−0.04	−0.02	−**0.16**	−**0.16**	−**0.18**	−0.01	−0.01	0.00
NA	−0.10	−0.12	−0.13	−0.10	−0.10	−0.09	**0.21**	**0.18**	**0.15**	**0.25**	**0.23**	**0.24**	0.10	0.12	0.09
Life Satis	0.07	0.06	0.05	0.11	0.11	0.12	−0.08	−0.06	−0.04	−0.09	−0.08	−0.10	−**0.13**	−**0.14**	−0.09

*Enh, Enhance; Div, Divert; Wor, Worsen; Inau, Inauthentic; Conc, Conceal; H, E, X, A, C, O, Honesty-Humility, Emotionality, Extraversion, Agreeableness, Conscientiousness, Opennes; SEA, self-emotional appraisal; OEA, others' emotional appraisal; UOE, use of emotion; ROE, regulation of emotion in self; Extr Aff Imp, Extrinsic Affect Improving; Extr Aff Wors, Extrinsic Affect Worsening; Intr Aff Imp, Intrinsic Affect Improving; Intr Aff Wors, Intrinsic Affect Worsening; PA, positive affect; NA, negative affect. N range 390–393, Correlations with p ≤ 0.01 shown in bold*.

**Table 13:** Bivariate Correlation comparisons for Sample 4.

	**Enh**	**Enh-SF**	**Enh-VSF**	**Div**	**Div-SF**	**Div-VSF**	**Wor**	**Wor-SF**	**Wor-VSF**	**Inau**	**Inau-SF**	**Inau-VSF**	**Conc**	**Conc-SF**	**Conc-VSF**
H	**0.19**	**0.18**	0.16	−0.06	−0.07	−0.08	−**0.42**	−**0.40**	−**0.38**	−**0.55**	−**0.48**	−**0.32**	0.09	0.06	0.09
E	**0.22**	**0.20**	**0.21**	0.10	0.08	0.10	−0.13	−0.15	−0.08	0.13	0.14	**0.22**	−0.15	−0.12	−0.09
X	**0.21**	0.17	0.17	**0.33**	**0.30**	**0.23**	0.04	0.06	0.06	−0.07	−0.09	−**0.17**	−**0.21**	−**0.21**	−0.16
A	**0.24**	**0.21**	**0.18**	**0.24**	**0.25**	**0.18**	−**0.45**	−**0.42**	−**0.44**	−**0.33**	−**0.36**	−**0.42**	**0.36**	**0.29**	**0.36**
C	0.14	0.14	0.16	0.05	0.04	0.03	−0.13	−0.09	−0.06	−0.13	−0.15	−0.11	−0.04	−0.05	−0.04
O	0.06	0.08	0.12	−0.02	−0.01	−0.06	−0.10	−0.12	−0.12	0.05	0.04	−0.05	−0.05	−0.05	−0.03
SEA	**0.18**	0.14	0.14	0.07	0.05	0.02	0.01	−0.01	0.01	−0.13	−0.14	−0.16	−0.13	−0.14	−0.09
OEA	**0.58**	**0.52**	**0.51**	**0.35**	**0.32**	**0.28**	−0.17	−0.19	−0.16	−0.16	−0.12	−0.06	0.14	0.16	**0.20**
UOE	**0.26**	**0.23**	**0.22**	**0.22**	**0.20**	0.13	−0.07	−0.05	−0.03	−0.09	−0.10	−0.13	−0.12	−0.12	−0.10
ROE	**0.17**	0.13	0.12	**0.23**	**0.24**	**0.22**	−0.08	−0.07	−0.08	−0.10	−0.09	−**0.18**	**0.18**	0.14	**0.18**
Emanage	**0.22**	**0.21**	**0.18**	0.08	0.06	0.04	**0.27**	**0.19**	0.19	0.12	0.13	0.10	−0.17	−0.15	−0.13
Relskills	**0.42**	**0.38**	**0.37**	**0.25**	**0.23**	**0.19**	−**0.35**	−**0.31**	−**0.25**	−**0.27**	−**0.26**	−**0.20**	0.09	0.06	0.13
Soccomp	**0.31**	**0.30**	**0.30**	**0.28**	**0.25**	**0.20**	0.01	0.02	0.02	−0.03	−0.02	−**0.07**	−**0.21**	−**0.20**	−0.16
Empathy	**0.53**	**0.49**	**0.48**	**0.29**	**0.27**	**0.21**	−**0.42**	−**0.39**	−**0.34**	−**0.30**	−**0.28**	−**0.26**	0.13	0.11	**0.19**
Extr Aff Imp	**0.49**	**0.43**	**0.41**	**0.41**	**0.37**	**0.32**	−0.08	−0.10	−0.09	−0.02	−0.02	−0.05	0.13	0.15	0.13
Extr Aff Wors	−0.15	−**0.20**	−**0.24**	−0.08	−0.08	−0.05	**0.42**	**0.39**	**0.36**	**0.33**	**0.30**	**0.29**	−0.16	−0.13	−0.13
Intr Aff Imp	**0.25**	**0.21**	**0.19**	**0.33**	**0.28**	**0.25**	−0.04	−0.04	−0.03	0.02	0.03	−0.02	−0.13	−0.13	−0.08
Intr Aff Wors	−0.10	−0.11	−0.13	−0.05	−0.05	−0.02	0.08	0.02	−0.02	**0.20**	**0.18**	**0.20**	0.10	0.11	0.11
PA	**0.24**	**0.19**	**0.19**	**0.30**	**0.31**	**0.28**	0.01	0.03	0.02	−0.05	−0.06	−0.10	−0.10	−0.11	−0.06
NA	−0.04	−0.06	−0.05	−0.08	−0.09	−0.07	0.12	0.07	0.07	**0.22**	**0.20**	**0.21**	−0.03	0.03	0.01
Life Satis	**0.18**	0.16	**0.18**	**0.20**	**0.19**	0.14	−0.08	−0.05	−0.04	−0.09	−0.10	−0.15	−**0.23**	−**0.25**	−**0.18**

*Enh, Enhance; Div, Divert; Wor, Worsen; Inau, Inauthentic; Conc, Conceal; H, E, X, A, C, O, Honesty-Humility, Emotionality, Extraversion, Agreeableness, Conscientiousness, Openness; SEA, self-emotional appraisal; OEA, others' emotional appraisal; UOE, use of emotion; ROE, regulation of emotion in self; Extr Aff Imp, Extrinsic Affect Improving; Extr Aff Wors, Extrinsic Affect Worsening, Intrinsic Aff Imp, Intrinsic Affect Improving; Intr Aff Wors, Intrinsic Affect Worsening; PA, positive affect; NA, negative affect. N range 222–223. Correlations with p ≤ 0.01 shown in bold*.

#### Sex differences in MEOS scores

Previous work has indicated a consistent sex difference in scores on the Worsen scale, with males scoring higher than females (Bacon and Regan, 2016) and with sex acting as a significant predictor when included as a predictor with personality and trait EI in regression models for this subscale score (Austin et al., 2014; Austin and Vahle, 2016). Sex differences in scores for all versions of the MEOS in Samples 3 and 4 were examined using *t*-tests, correcting for multiple comparisons. The only significant results were higher scores for males on Worsen for the MEOS, MEOS-SF and MEOS-VSF in Sample 3 [*t*_(389)_, 3.85, *p* < 0.001; *t*_(391)_, 4.40, *p* < 0.001; *t*_(391)_, 4.80, *p* < 0.001] and on the MEOS-SF and MEOS-VSF for Sample 4 [*t*_(224)_ = 3.53, *p* = 0.001; *t*_(224)_ = 3.61, *p* < 0.001].

#### MEOS associations with the EROS, WLEIS, PANAS, and life satisfaction

The associations of the MEOS with the other study scales showed the expected strong correlations of Enhance and Divert with the conceptually-related Extrinsic Affect Improving scale, and of Worsen and Inauthentic with the conceptually-related Extrinsic Affect Worsening scale. For the WLEIS, significant associations were positive for Enhance and Divert and negative for Worsen and Inauthentic, with the associations of Enhance and Divert with OEA scores being the strongest. Enhance and Divert were also strongly positively correlated with PA, with Worsen and Inauthentic more weakly associated with NA. Enhance and Divert also showed weak positive associations with life satisfaction.

### Discussion

The results showed satisfactory or good internal reliabilities for the MEOS-SF and MEOS-VSF subscale scores in both samples and a pattern of similar correlations with scores on other measures across all MEOS versions. Test-retest reliabilities of the short form scores were good in Sample 4 but lower in Sample 3. Low test-retest reliability coefficients were also found for subscale scores of the conceptually similar EROS scale and for NA scores in this sample; it is unclear why this sample showed low retest stability across several measures. Examination of sex differences showed that males scored higher than females on the Worsen subscale of the short forms; this difference is consistent with previous results for the full-length MEOS.

The associations of the MEOS with the EROS, WLEIS and PANAS are of interest as they have not been examined previously. Associations of the EROS extrinsic subscales with the core MEOS factors showed the expected pattern of strong associations of Enhance and Divert with Extrinsic Affect Improving, and of Worsen and Inauthentic with Extrinsic Affect Worsening. This aligns with the conceptual relationships of the MEOS core subscales and the EROS extrinsic subscales. The MEOS associations with the WLEIS subscales were consistent with the pattern of positive associations with trait EI for Enhance and Divert and negative associations for Worsen and Inauthentic found in previous work (e.g., Austin and O'Donnell, 2013), with the larger associations for Enhance and Divert with OEA being consistent with this WLEIS facet covering perceiving and understanding others' emotions (Wong and Law, 2002), although this pattern did not extend to Worsen and Inauthentic. The associations of the core MEOS factors with PA and NA parallel previous results showing that using interpersonal affect regulation for improving or worsening others' moods has congruent associations with affective well-being (Niven et al., 2012).

## General discussion

In these two studies two short forms of the MEOS were developed and examined using both previously-available data and two new samples. The results showed that the MEOS-SF (six items per subscale) and the MEOS-VSF (four items per subscale) scores were reliable and also displayed similar validity coefficients with a range of personality, trait EI and well-being measures to those of the full-length MEOS. New results cross-validating the MEOS with the interpersonal scales of the EROS (Niven et al., 2011), which also assess interpersonal emotion management, were also presented.

The limitations of the present work include the use of predominantly female student samples. Further work on all MEOS versions in samples more representative of the general population is desirable, including the study of age differences in interpersonal emotion management.

The present work was also limited by only including self-report scales. One important way to extend the results would be to examine how MEOS scores are associated with behavioral measures of interpersonal emotion management, for example by examining the effects of these scores as potential moderators of approaches to regulating another's emotions in laboratory social interaction scenarios (Andrade and Ho, 2009), or in realistic social interactions. Given the positioning of interpersonal emotion management within the social domain, the creation and use of peer-report MEOS versions would also be of considerable interest. An example of an area where peer reports could be informative is in the examination of sex differences in the way in which interpersonal emotional management is used in social interactions (Bacon and Regan, 2016).

The good psychometric properties of the MEOS short forms mean that these scales are suitable for use in contexts where a brief assessment of interpersonal emotion management is required. All versions of the MEOS allow examination of the manipulative as well as the helpful/prosocial aspects of interpersonal emotion management, so are particularly relevant to studies where the “dark side” facets of trait EI, as well as its more prosocial aspects, are of interest. The short forms should be particularly useful in some of the areas for future research highlighted above, since short scales are of practical utility for behavioral and longitudinal studies, and for studies using peer-reports. The specific choice of the MEOS-SF or MEOS-VSF, assuming a study design for which the full MEOS is not suitable, depends on a trade-off between the better reliability and domain coverage of the MEOS-SF against the smaller number of items per subscale, and hence demand on participants, for the MEOS-VSF. The brevity of the MEOS-VSF means that it would be particularly suitable for studies using experience sampling (e.g., Catterson et al., 2017) and similar repeat-measure designs, where there is a requirement to complete the same scale on multiple occasions. For single surveys where the requirement for a very short scale is less pressing, the MEOS-SF would generally be more suitable.

## Data availability

The raw data supporting the conclusions of this manuscript will be made available by the authors, without undue reservation, to any qualified researcher.

## Author contributions

EA: Project design and administration, survey creation, data collection, data preparation and coding, data analysis, writing of manuscript; DS: Project design and administration, manuscript revision; MS: Project design and administration, survey creation, data collection, data preparation and coding, manuscript revision.

### Conflict of interest statement

The authors declare that the research was conducted in the absence of any commercial or financial relationships that could be construed as a potential conflict of interest.
